# Rapid Antigen Detection Testing for Group A Streptococcus as an Adjunct for Early Diagnosis of Necrotizing Soft Tissue Infection: A Case Series

**DOI:** 10.7759/cureus.113331

**Published:** 2026-07-24

**Authors:** Kaiho Hirata, Naoki Shida, Haruka Tsuji, Takuyo Chiba, Takashi Shiga

**Affiliations:** 1 Department of Emergency Medicine, AdventHealth Tampa, Tampa, USA; 2 Department of Emergency Medicine, International University of Health and Welfare Narita Hospital, Narita, JPN

**Keywords:** early diagnosis and treatment, group a streptococcus, necrotizing fasciitis, necrotizing soft tissue infection (nsti), rapid antigen detection test

## Abstract

Necrotizing soft tissue infection (NSTI) is a rapidly progressive, life-threatening soft-tissue infection most commonly caused by group A Streptococcus (GAS). Although early diagnosis and prompt surgical intervention are crucial, the initial presentation is often nonspecific, which can occasionally delay diagnosis and surgical intervention. We report three cases of NSTI caused by GAS in which rapid antigen detection tests (RADTs), in conjunction with imaging and surgical exploration findings, were helpful in the diagnosis of NSTI: a 47-year-old man presented with left groin swelling and pain (Case 1); a 72-year-old man with right thigh erythema and tenderness (Case 2); and a 63-year-old man with right lower leg redness and pus discharge (Case 3). All three patients had elevated levels of inflammatory markers, and imaging revealed subcutaneous fat stranding. RADTs performed on the wound or tissue samples were positive in all cases prior to culture confirmation. Each patient underwent prompt surgical debridement combined with empirical broad-spectrum antibiotics. In Case 3, the Laboratory Risk Indicator for Necrotizing Fasciitis (LRINEC) score was a false negative, demonstrating the limitations of laboratory-based scoring in early NSTI. However, a positive RADT result, in conjunction with subcutaneous fat stranding observed on computed tomography scan, enabled timely surgical intervention. This study suggests that RADTs, when combined with clinical assessment and imaging, may serve as valuable adjuncts for early diagnosis and management of NSTI.

## Introduction

Necrotizing soft tissue infection (NSTI) is a rapidly progressive, life-threatening soft-tissue infection most commonly caused by group A Streptococcus (GAS) [1-3]. Although a slight reduction in mortality has been reported, NSTI remains associated with a high mortality rate [4]. Early diagnosis and treatment are crucial but can be challenging owing to the variable initial presentation. Causative organisms are typically identified by tissue or blood culture, although this process can be delayed by several days.

The rapid antigen detection test (RADT) for GAS was originally developed for acute pharyngitis. Although several case reports have described the successful use of RADTs from patients with NSTI, standardized evaluation of their diagnostic utility is lacking [5,6].

Imaging studies are crucial in the early diagnosis of NSTI; among these modalities, computed tomography (CT) is a rapid and accessible tool. Characteristic findings include perifascial fluid collection and fascial thickening [7,8]. Although imaging studies assist in the diagnosis of NSTI, definitive diagnosis requires surgical exploration and direct inspection of the lesions, such as the finger test. Characteristic findings of NSTI include dishwater lack of bleeding and necrotic tissue with little resistance to blunt finger dissection [9,10]. However, these findings are subjective and may vary depending on the physician’s experience. Therefore, an objective diagnostic adjunct such as RADT performed on wound or tissue specimens obtained during surgical exploration, when combined with clinical, imaging, and intraoperative findings, may further support the diagnosis of NSTI.

We report three cases of GAS-induced NSTI, in which RADT for GAS, combined with imaging studies, facilitated early diagnosis. This case series aims to support the clinical utility of RADT in the diagnosis of NSTI.

## Case presentation

Case 1

**Figure 1:**
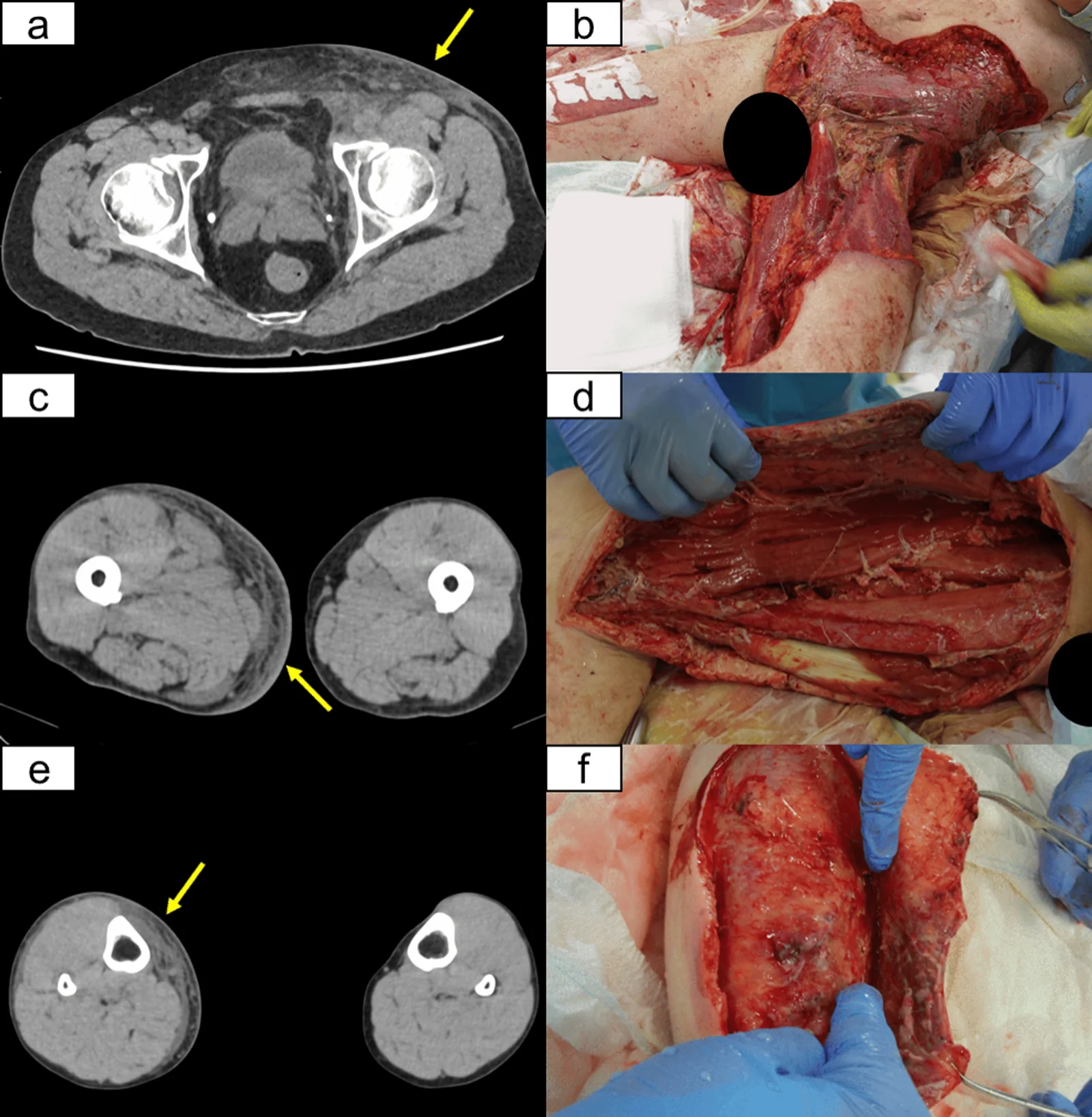
Findings of CT and wounds during emergent debridement on day 1 in each case. (1) CT findings of Case 1 (a). Subcutaneous fat stranding from the anterior to medial aspect of the left thigh. (b) Findings of the wound during the debridement in Case 1. Necrotic subcutaneous tissue, muscle, and fascia were incised across the left flank, bilateral lower abdomen, left anterior thigh, and bilateral spermatic cords and testes. (2) CT findings of Case 2 (c). Subcutaneous fat stranding and marked fascial thickening in the right thigh without gas. (d) Findings of the wound during the debridement in Case 2. Necrotic subcutaneous tissue, muscle, and fascia extending from the right proximal thigh to the right popliteal fossa were incised. (3) CT findings of Case 3 (e). Subcutaneous fat stranding and edema in the anteromedial right lower leg. (f) Findings of the wound during the debridement in Case 3. Necrotic subcutaneous tissue, muscle, and fascia were incised in the anterior aspect of the right lower leg.

A 47-year-old man with a history of hypertension and atopic dermatitis visited our hospital on day 1, complaining of left groin pain. His symptoms began with fever four days earlier, followed by left groin swelling three days earlier. Upon arrival, his vital signs were as follows: temperature, 36.9°C; blood pressure, 92/55 mmHg; heart rate, 99 beats/min; respiratory rate, 24 /min; and oxygen saturation, 97% on room air. Physical examination revealed a reddish-purple discoloration extending from the left inguinal region to the left anterior thigh, with local warmth, vesicles, and severe pain. Multiple erythematous macules (0.5-1 mm in diameter) were scattered over the lower abdomen with mild pruritus. Laboratory tests revealed elevated inflammatory markers, including a white blood cell count (WBC) of 9270/µL and a C-reactive protein (CRP) level of 40.0 mg/dL with high serum creatinine of 5.57, high total bilirubin of 7.5, and low creatine kinase of 37 (Table 1). Contrast-enhanced CT revealed subcutaneous fat stranding from the midline of the lower abdomen, extending to the left side, left inguinal region, and the anterior-to-medial aspect of the left thigh (Figure 1a). Fat stranding extended to the left retroperitoneal space at the level of the bifurcation of the internal and external iliac arteries. Emergency bedside surgical exploration revealed necrotic subcutaneous tissue, muscles, and fascia of the left flank, bilateral lower abdomen, left anterior thigh, and bilateral spermatic cords and testes (Figure 1b).

**Table 1:** Laboratory data on admission. PT: prothrombin time; INR: international normalized ratio; APTT: activated partial thromboplastin time Note: * Laboratory value above reference range; ** Laboratory value below reference range.

Variable	Case 1	Case 2	Case 3	Reference range
White blood cell count (per μL)	9,270*	22,580*	15,930*	3300-8600
Neutrophil (%)	90*	94.2*	88.3*	38.5-80.5
Red blood cell count (10^6 per μL)	5.96*	3.21**	5.18*	4.35-5.55
Hemoglobin (g/dL)	16.7	9.8**	15.3	13.7-16.8
Hematocrit (%)	50.3*	28.9**	43.9	40.7-50.1
Platelet count (per μL)	189,000	220,000	222,000	158,000-348,000
PT-INR	1.46*	1.04	1.00	0.90-1.10
APTT (sec)	40.9*	33.4	26.8	25.0-40.0
Fibrinogen (mg/dL)	888*	705*	573*	160.0-350.0
Blood urea nitrogen (mg/dL)	44.8*	38.5*	15.6	8-20
Creatinine (mg/dL)	5.57*	1.37*	1.32*	0.65-1.07
Albumin (g/dL)	3.5**	2.4**	4.2	4.1-5.1
Aspartate aminotransferase (U/L)	24	38*	116*	13-30
Alanine aminotransferase (U/L)	24	34	130*	10-42
Total bilirubin (mg/dL)	7.5*	6.0*	2.8*	0.4-1.5
C-reactive protein (mg/dL)	40.26*	36.66*	14.49*	0.0-0.14
Creatine kinase (U/L)	37**	258*	64	59-248
Na (mmol/L)	135**	133**	137**	138-145
K (mmol/L)	3.3**	3.6	3.6	3.6-4.8
Cl (mmol/L)	92**	99**	99**	101-108
Ca (mg/dL)	8.8	7.5**	9.0	8.8-10.1
P (mg/dL)	2.3**	1.2**	1.3**	2.7-4.6
Glucose (mg/dL)	191*	191*	151*	73-109
HbA1c (%)	5.4	6.0	5.9	4.9-6.0

A RADT for GAS from the wound was positive, and wound culture later returned positive for GAS. Accordingly, the patient was diagnosed with NSTI and received bedside surgical debridement. Empirical antibiotic therapy with clindamycin, meropenem, and vancomycin was initiated.

On day 5, despite initial debridement, the patient developed a fever with elevated inflammatory markers. A follow-up CT scan on day 6 revealed extension of the lesion into the left retroperitoneal space. In addition to debridement of the left retroperitoneal lesion on day 7, additional debridement was performed in the operating room on days 9, 12, 15, and 23. Subsequently, the patient’s fever subsided, and inflammatory markers gradually improved. Vacuum-assisted closure (VAC) therapy was initiated on day 29 and continued until day 82. Skin grafting was performed on postoperative days 62 and 74. On day 161, the patient was transferred to a rehabilitation hospital.

Case 2

A 72-year-old man with a history of benign prostatic hyperplasia was referred to our hospital on day 1 with fever, right thigh pain, and suspected NSTI. His symptoms began four days earlier. Upon arrival, his vital signs were as follows: temperature, 37.8°C; blood pressure, 110/80 mmHg; heart rate, 110 beats/min; and oxygen saturation, 95% on room air. Physical examination revealed erythema, swelling, and tenderness in the right posterior thigh.

Laboratory tests revealed elevated inflammatory markers, including a WBC of 22,580/µL and a CRP of 36.7 mg/dL with high total bilirubin of 6.0 (Table 1). Contrast-enhanced CT revealed subcutaneous fat stranding, marked fascial thickening, and perifascial fluid collection in the right thigh without gas (Figure 1c). During the finger test, the subcutaneous tissue separated easily and “dishwater” discharge was observed with minimal bleeding. A RADT for GAS from the wound was positive, and wound culture later returned positive for GAS. Accordingly, the patient was diagnosed with NSTI and received bedside surgical debridement. During the surgical debridement, the fascia extending from the right proximal thigh to the right popliteal fossa was incised (Figure 1d). Empiric antibiotic therapy with clindamycin, meropenem, and vancomycin was initiated.

On day 3, the blood culture obtained on day 1 tested positive for GAS. VAC therapy was initiated on day 6; however, the wound showed signs of infection on day 10, leading to the discontinuation of VAC therapy. Additional debridement was performed in the operating room on day 23, with VAC therapy resumed the same day. The patient underwent skin grafting on day 42, completed VAC therapy on day 48, and was discharged on day 69.

Case 3

A 63-year-old man with a medical history of hypertension, hyperlipidemia, and prostate cancer status post surgery one year prior presented with right lower leg redness and swelling on day 1. He had injured his right lower leg six days earlier after falling down stairs and had purulent discharge from the wound for several days. Upon arrival, his vital signs were as follows: blood pressure, 149/99 mmHg; heart rate, 107 beats/min; respiratory rate, 16/min; oxygen saturation, 97% on room air; and temperature, 37.8°C. Physical examination revealed a 3 cm crusted lesion with white milky fluid discharge, accompanied by warmth and erythema surrounding the right lower leg.

Laboratory tests revealed elevated inflammatory markers, including a WBC of 15,930/µL and a CRP level of 14.49 mg/dL (Table 1). Contrast-enhanced CT revealed subcutaneous fat stranding and edema surrounding the wound in the anteromedial right lower leg (Figure 1e). During the emergent bedside surgical exploration, necrotic subcutaneous tissue, muscle, and fascia in the anterior aspect of the right lower leg were observed (Figure 1f). A RADT for GAS from the wound was positive, and wound culture later returned positive for GAS and methicillin-sensitive* Staphylococcus aureus*. The patient was diagnosed with NSTI and received bedside surgical debridement. Empirical antibiotic therapy was initiated with piperacillin/tazobactam, clindamycin, and vancomycin.

The patient underwent additional debridement in the operating room on day 9, initiation of VAC therapy on day 11, skin grafting on day 22, completion of VAC therapy on day 31, and was discharged on day 43 of hospitalization.

A summary of the clinical course of each case is presented in Table 2.

**Table 2:** Summary of the clinical course of each patient. LRINEC: Laboratory Risk Indicator for Necrotizing Fasciitis

Parameter	Case 1	Case 2	Case 3
Age	47	72	63
Sex	Male	Male	Male
Past medical history	Hypertension, atopic dermatitis	Benign prostatic hyperplasia	Hypertension, hyperlipidemia, prostate cancer
First symptom	Fever	Fever, right thigh pain	Injury to the right lower leg after falling down stairs
Days from first symptom to admission	4 days	4 days	6 days
LRINEC score on day 1	7	10	1
Affected lesion	Left flank, bilateral lower abdomen, left anterior thigh, bilateral spermatic cords and testes	Right posterior thigh	Right anteromedial lower leg
Days from admission to discharge	161 days	69 days	43 days

## Discussion

NSTI is a rapidly progressing, life-threatening soft-tissue infection necessitating early diagnosis and surgical intervention [11]. However, its initial clinical presentation is often nonspecific, making differentiation from other soft-tissue infections (e.g., cellulitis) difficult [12,13]. Although surgical exploration remains the gold standard for diagnosing NSTI, characteristic intraoperative findings are subjective, and it can be difficult to make a diagnosis even for experienced surgeons [14,15]. In this case series, we report three cases of GAS-induced NSTI in which GAS RADTs contributed to the diagnosis. In these cases, although the clinical, imaging, and intraoperative findings were already highly concerning for NSTI, the positive RADT result provided microbiological support for GAS involvement and reinforced the diagnosis of GAS-associated NSTI. Only a limited number of reports have described the usefulness of RADTs for GAS in NSTI cases, and their effectiveness remains poorly established [5,6]. Our findings suggest that RADT for GAS may serve as a valuable diagnostic adjunct in NSTI cases. Importantly, RADT should be considered an adjunctive diagnostic tool and should never delay urgent surgical exploration when NSTI is clinically suspected.

In Cases 1 and 2, the LRINEC score was positive, supporting the diagnosis [16]. In these highly suspect cases, even though RADT provides microbiological support for GAS involvement, its usefulness is limited. However, in Case 3, the LRINEC score was a false negative. The diagnosis was established through an exploratory incision and a positive finger test. In this patient, a positive RADT of the discharge obtained before and during the exploratory incision facilitated prompt surgical intervention and provided additional diagnostic support, respectively. Because the LRINEC score relies on laboratory parameters, its sensitivity may be reduced in early-stage disease when systemic inflammatory changes are minimal [17,18]. Therefore, combining the RADT, which directly detects the causative pathogen, with imaging and intraoperative findings could serve as a useful complement to conventional scoring systems.

This study is limited by the small number of cases, and the findings are not generalizable to non-GAS NSTI. When RADT for GAS is used as a part of the NSTI diagnosis, clinicians should remain mindful that, although GAS is the most common causative organism, NSTI may also be caused by pathogens other than GAS. Moreover, RADT sensitivity may vary by sampling site and infection stage. Future studies with larger cohorts are needed to evaluate the diagnostic performance of the RADT alone and in combination with other noninvasive diagnostic tools, including the LRINEC score, ultrasonography, and CT [19]. In addition, a positive RADT identifies GAS but does not distinguish NSTI from non-necrotizing GAS soft tissue infections. Therefore, RADT findings must always be interpreted within the overall clinical and radiological context.

## Conclusions

Because of the nonspecific initial presentation and the subjective nature of intraoperative findings during surgical exploration, NSTI remains challenging to diagnose early. Even though the evidence is limited by the small number of cases, our case series generates the hypothesis that RADT for GAS, when combined with clinical assessment, imaging, and surgical exploration findings, may have a potential role in the diagnosis of GAS-induced NSTI. Larger prospective studies are necessary to determine its diagnostic accuracy and clinical usefulness before broader clinical recommendations can be made. Of note, although RADT positivity suggests microbiological evidence of GAS involvement in the affected area, RADT alone does not suggest the NSTI diagnosis because RADT would be positive even in non-necrotizing soft tissue infection by GAS.
